# 

**Published:** 1902-06-14

**Authors:** 


### NOTICE TO CORRESPONDENTS.

All MSS., Letters, Books for Review, and other matters intended for the Editor should be addressed The Editor, "The Hospital," The Hospital Building, 28 & 29 Southampton Street, Strand, W.C.
The Editor cannot undertake to return rejected MS., even when accompanied by stamped directed envelope.

### Notice to Advertisers and Subscribers.

All Advertisements, Orders for Copies of The Hospital, and Business Communications should be addressed to **THE MANAGER, (Not to the Editor),** The Hospital, 28 & 29 Southampton Street, Strand, London, W.C.

The Cost of Subscription, post free, is as follows.—Per week, 2½d.; per quarter, 2s. 8d.; per half-year, 5s. 3d.; per annum, 10s. 6d. Foreign:—Per annum, 15s. 2d., payable, in all cases, in advance.

**NOTICE.—Vol. XXXI. of "The Hospital" (October 1901, to March 1902), in handsome cloth binding is now on sale at the office of this Journal, price, 7s. 6d. Cases for binding the Numbers contained in this Volume 1s. 6d. Index to Vol. XXXI., 2½d., post free.**
**The Monthly Part for June is now ready. Price 6d., by post 8d.**

### The Student of Medicine.

### Appointments.

M. B. Arnold, M.B., Ch.B.Vict., appointed House Physician to the Manchester Royal Infirmary. Claude C. Claremont, M.D., appointed Physician to the Royal Portsmouth and Gosport Hospital. Thomas S. Collin, L.S.A., appointed District Medical Officer of the Howden Union. Ptolemy Augustus Culmer, M.R.C.S.Eng., L.R.C.P.Lond., L.S.A., appointed Medical Officer and Public Vaccinator to No. 2 District, Ryemill Union. W. J. Cowden, M.D.R.U.I., M.Ch, appointed Certifying Factory Surgeon for the Dromore District, County Down. J. W. H. Eyre, M.D., appointed Bacteriologist to Guy's Hospital Frederick Anthony Floyer, B.A., M.B.Camb., M.R.C.S.Eng., L.S.A.Lond., Civil Surgeon attached to Connaught Hospital, Aldershot, appointed a Government Medical Officer British Central Africa, to proceed to Zomba, B.C.A. W. P. Gowland, M.R.C.S., L.R.C.P.Lond., appointed House Surgeon to the Manchester Royal Infirmary. R. B. Greaves, B.S., M.B., appointed Medical Officer for Third District, and Public Vaccinator for First District of Ecclesall Bierlow Union.

